# Tandem autologous hematopoietic stem cell transplantation in multiple myeloma: A historical perspective and current challenges

**DOI:** 10.1007/s00277-025-06563-z

**Published:** 2025-09-15

**Authors:** Xueting Wang, Yushan Cui, Yaomei Wang, Baijun Fang

**Affiliations:** Department of Hematology, Henan Institute of Hematology, The Affiliated Cancer Hospital of Zhengzhou University & Henan Cancer Hospital, Zhengzhou, 450008 China

**Keywords:** High-risk multiple myeloma, Multiple myeloma, Tandem hematopoietic stem cell transplantation, Allogeneic hematopoietic stem cell transplantation

## Abstract

Multiple myeloma (MM) is a heterogeneous and relapse-prone hematologic malignancy that remains incurable. For newly diagnosed patients aged 70 years or younger, who are eligible for transplantation, autologous hematopoietic stem cell transplantation (auto-HSCT) is the preferred first-line treatment. In patients with high-risk multiple myeloma (HRMM), some studies have demonstrated that tandem auto-HSCT provides notable benefits over single auto-HSCT, particularly in extending progression-free survival (PFS) and overall survival (OS). Although allogeneic hematopoietic stem cell transplantation (allo-HSCT) currently offers the only potential for long-term cure in MM, its application is limited by high transplant-related mortality (TRM) and the risk of graft-versus-host disease (GVHD). In recent years, the emergence of novel therapies, including proteasome inhibitors, immunomodulatory drugs, monoclonal antibodies, and chimeric antigen receptor T-cell (CAR-T) therapy, has posed new challenges to the role of tandem auto-HSCT in MM treatment. This review aims to critically examine the efficacy differences between tandem and single auto-HSCT, and sequential allo-HSCT following auto-HSCT. Furthermore, it will rigorously evaluate the role and challenges of tandem auto-HSCT within the evolving therapeutic landscape.

## Introduction

Multiple myeloma (MM) is a malignant hematologic disorder characterized by the abnormal proliferation of clonal plasma cells, accounting for approximately 10% of hematologic malignancies and 1% of all cancers [1]. Despite significant therapeutic progress in recent years, MM remains incurable and exhibits a high relapse rate.

Since the 1980s, autologous hematopoietic stem cell transplantation (auto-HSCT) demonstrates substantial clinical efficacy in treating MM and remains a cornerstone of therapy [2, 3]. Additional strategies, including allo-HSCT, sequential auto-HSCT followed by allo-HSCT, and tandem auto-HSCT, have also been integrated into treatment protocols for MM.

The introduction of proteasome inhibitors (PIs) (e.g., ixazomib, bortezomib, carfilzomib) and immunomodulatory drugs (IMiDs) (e.g., lenalidomide, pomalidomide) has led to significant advances in induction therapy for MM. Three-drug regimens based on lenalidomide and bortezomib (such as RVd) are widely used as standard initial therapy for newly diagnosed multiple myeloma (NDMM). In patients with high-risk multiple myeloma (HRMM), four-drug combinations incorporating a CD38 monoclonal antibody (e.g., D-RVd or D-KRd) have shown promising efficacy and are increasingly considered as a preferred frontline approach [4, 5].

Several studies indicate that induction therapy with PIs and/or IMiDs followed by auto-HSCT substantially improves PFS [2, 6–8] and OS [2, 7, 8]. This underscores the continued importance of auto-HSCT in the current treatment landscape. Four-drug regimens not only deepen responses in patients with HRMM, but also significantly increase the rate of minimal residual disease (MRD) negativity prior to auto-HSCT in standard-risk patients, thereby laying a foundation for subsequent consolidation therapy [9]. Against this backdrop, single auto-HSCT remains a cornerstone strategy for transplant-eligible patients. However, the necessity of tandem auto-HSCT—particularly its potential to further improve outcomes in HRMM patients—remains a subject of ongoing debate.

Notably, the rapid advancement of immunotherapy has prompted a re-evaluation of its impact on transplant strategies. Innovative approaches—including CD38 monoclonal antibodies (such as daratumumab), antibody–drug conjugates (ADCs) targeting B-cell maturation antigen (BCMA, such as belantamab mafodotin), and chimeric antigen receptor T-cell (CAR-T) therapies—have markedly improved long-term survival in patients with MM [10]. Currently, multiple studies are investigating the integration of these novel therapies with conventional transplantation approaches to optimize outcomes, particularly in high-risk patients.

This review examines recent advancements in tandem auto-HSCT for MM and assesses its efficacy compared to single auto-HSCT and sequential auto-HSCT followed by allo-HSCT. The objective is to refine the role of tandem transplantation within contemporary treatment strategies and to offer insights that can inform future clinical practices and research directions.

## Tandem auto-HSCT

Tandem autologous stem cell transplantation (tandem auto-HSCT) refers to a treatment strategy in which a second planned autologous transplant is performed within six months following the initial transplant. This approach involves sequential high-dose chemotherapy combined with consecutive stem cell reinfusion, aiming to achieve deeper remission by reducing MRD, to prolong the duration of remission, and to potentially improve OS [11].

For transplant-eligible patients who have not yet undergone autologous transplantation, initial auto-HSCT should be prioritized. In patients who relapse after a first auto-HSCT, salvage autologous transplantation may be considered; however, its efficacy largely depends on the duration of remission following the initial transplant. In those previously treated with conventional regimens such as bortezomib and lenalidomide, a remission duration exceeding 18 months is generally required. In contrast, for patients treated with more modern approaches—such as CD38 monoclonal antibody–based quadruplet induction and maintenance therapy—a longer remission period of over 36 months is recommended to maximize the benefit of salvage auto-HSCT [12]. In cases of shorter remission, alternative strategies such as targeted therapy or cellular therapy may offer greater advantages.

Recent data from the GMMG ReLApsE trial [13] further support this view. In relapsed and/or refractory multiple myeloma (RRMM) patients previously treated with frontline auto-HSCT, salvage high-dose chemotherapy followed by auto-HSCT and lenalidomide maintenance showed no significant survival advantage over continuous Rd (lenalidomide and dexamethasone). After a median follow-up of 99 months, PFS was 20.5 vs. 19.3 months (HR = 0.98, *p* = 0.9), and OS was 67.1 vs. 62.7 months (HR = 0.89, *p* = 0.44). These findings reinforce the notion that when novel agents such as lenalidomide are readily accessible, the clinical value of salvage auto-HSCT may be limited, and its use should be considered more selectively, particularly in resource-rich settings.

### Definition of HRMM

**Fig. 1:**
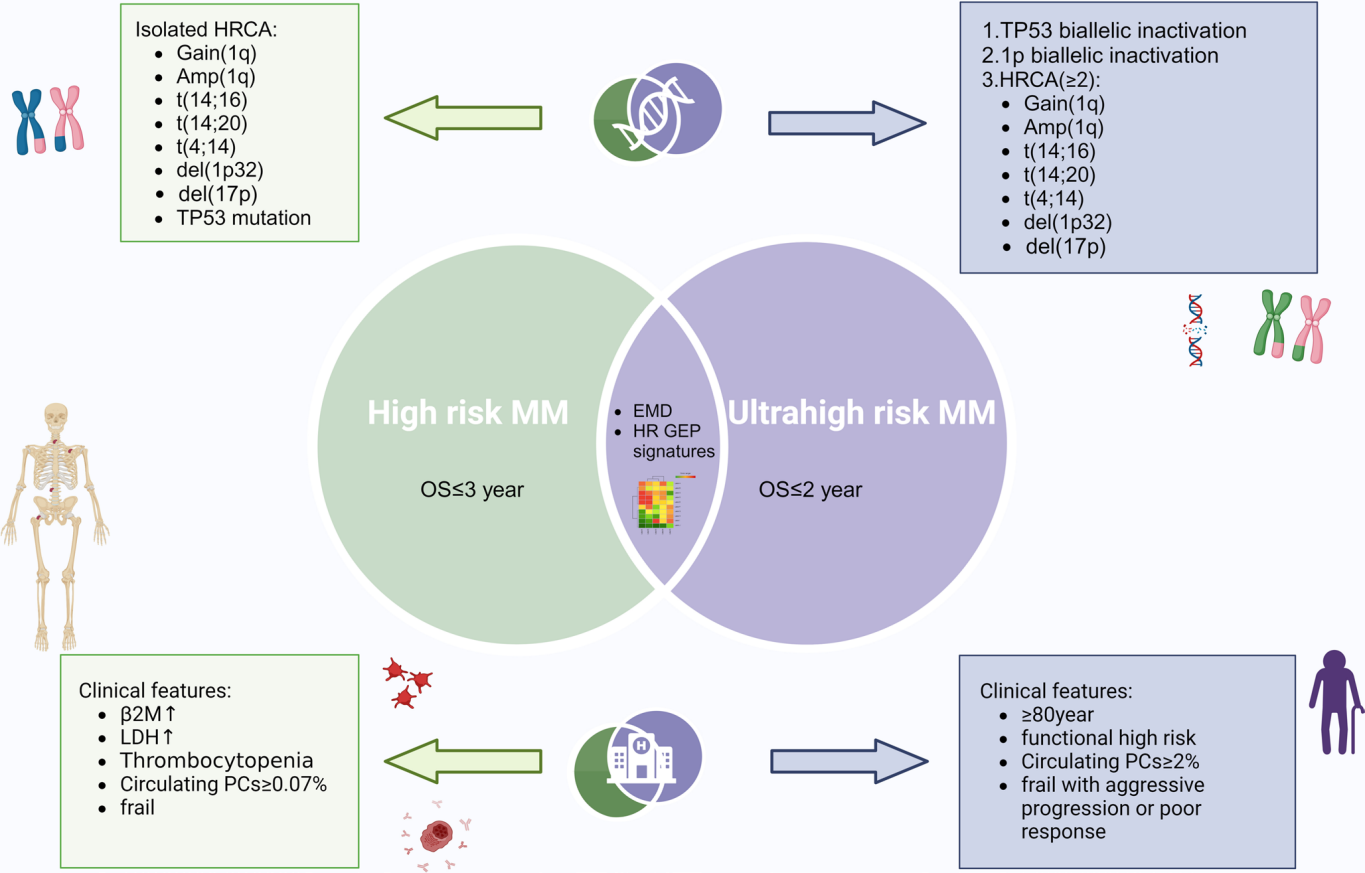
Risk stratification of high-risk and ultra-high-risk multiple myeloma. Abbreviations: AMP: Amplification; del: deletion; EMD: Extramedullary Disease; HRCA: High-Risk Cytogenetic Abnormalities; HR GEP: High-Risk Gene Expression Profiling; LDH: Lactate Dehydrogenase; MM: Multiple myeloma; OS: Overall Survival; PCs: Plasma Cells; t: translocation; TP53: Tumor Protein 53; β2M: β2-microglobulin. Created with BioRender.com

The definition of HRMM continues to evolve. With advancements in genomic technologies and ongoing refinement of risk assessment systems, stratification of various high-risk subtypes has become increasingly precise. In most clinical studies, HRMM is typically defined by specific cytogenetic abnormalities. Recently, the Consensus Genomic Staging (CGS) framework redefined high-risk cytogenetic features associated with HRMM. According to CGS, high-risk criteria include any of the following: deletion of the short arm of chromosome 17 (del(17p) with a clonal burden > 20% and/or TP53 mutation; IgH translocations such as t(4;14), t(14;16), or t(14;20) co-occurring with 1q21 amplification and/or 1p32 deletion; monoallelic del(1p32) combined with 1q21 amplification; or biallelic del(1p32) [14]. In addition, elevated β2-microglobulin (β2-MG) levels (≥ 5.5 mg/L) in the context of normal serum creatinine (< 1.2 mg/dL), as well as clinical features such as thrombocytopenia or an increased proportion of circulating plasma cells in peripheral blood, may also indicate an underlying high-risk biological profile [14, 15] (Fig. 1).

However, in clinical practice, a subset of patients without conventional high-risk genomic markers may still exhibit aggressive disease behavior, such as resistance to induction therapy or relapse within 12 months following initial treatment [16–19]. These patients are generally classified as having FHRMM. Although this category lacks a standardized definition and is not yet widely incorporated into risk stratification systems used in clinical trials, it is increasingly recognized in therapeutic decision-making. Genomic analyses have revealed that this subtype is frequently associated with molecular features such as aberrations in the IL-6/JAK/STAT3 signaling pathway, dysregulation of mitotic checkpoint control, and defects in the DNA damage response [20]. Evidence suggests that the combination of the DRd regimen (daratumumab, lenalidomide, and dexamethasone) with auto-HSCT can induce high rates of MRD negativity and durable disease control in patients with FHRMM [21], indicating that this intensified treatment strategy offers favorable clinical prospects for the FHR population.

Ultra-high-risk multiple myeloma (uHRMM) represents a particularly aggressive subtype of the disease. It is typically defined by the presence of two or more high-risk cytogenetic abnormalities—commonly referred to as “double-hit” or “triple-hit” features—including biallelic inactivation of TP53, biallelic deletion of 1p, and amplification of 1q. In many cases, this genomic profile is also accompanied by ≥ 2% CPCs in peripheral blood. Patients with uHRMM generally experience markedly shortened survival and a significantly elevated risk of early relapse [22, 23](Fig. 1). From a clinical progression standpoint, HRMM is generally characterized by disease progression or death occurring within 36 to 60 months after diagnosis, whereas patients with uHRMM typically experience progression or death within 24 to 36 months [10, 24]. The OptiMMum trial enrolled 107 patients with uHRMM to evaluate the efficacy of an intensified treatment strategy consisting of daratumumab plus CVRd (cyclophosphamide, bortezomib, lenalidomide, and dexamethasone) as induction, followed by high-dose melphalan and auto-HSCT. At a median follow-up of 22.2 months, the overall response rate (ORR) at day 100 post-auto-HSCT reached 83%, with a complete response (CR) rate of 47% and a MRD negativity rate of 64% [25].

Notably, patients harboring two or more high-risk genetic abnormalities—termed “double-hit” (HR = 2.28, 95% CI: 1.76–2.95) [22] or “triple-hit” myeloma (median OS < 22 months) [26]—often exhibit genomic instability events such as biallelic TP53 inactivation or 1p32 deletion, further worsening prognosis [22, 23]. Importantly, the mSMART 4.0 risk stratification system has eliminated the separate classification of “triple-hit” myeloma and instead defines double-hit myeloma as the presence of two or more high-risk cytogenetic abnormalities. This subgroup is associated with extremely poor prognosis and presents significant challenges in clinical management [12].

In summary, risk stratification in HRMM has evolved from a static genomics-based model toward an integrated approach that incorporates dynamic disease progression characteristics. Achieving deep and sustained MRD negativity remains the primary therapeutic goal for patients with HRMM, FHRMM, and uHRMM. For transplant-eligible HRMM patients, intensified induction regimens based on carfilzomib—such as KRd (carfilzomib, lenalidomide, and dexamethasone) [27]—or quadruplet combinations incorporating anti-CD38 monoclonal antibodies (e.g., Dara-KRd or Dara-VRd) [4, 5], followed by auto-HSCT, have been shown to significantly improve MRD negativity rates and long-term survival outcomes [28]. In the consolidation and maintenance phases, combining anti-CD38 monoclonal antibodies with IMiDs and PIs is recommended to sustain MRD negativity. For patients with uHRMM, treatment strategies need to be more intensive and individualized. On the basis of auto-HSCT, induction with a four-drug regimen followed by intensified consolidation is recommended. Although novel therapies are rapidly emerging, auto-HSCT remains a cornerstone of current treatment. Some studies suggest that tandem auto-HSCT may offer benefits in delaying disease progression; however, its clinical value in uHRMM remains uncertain and requires further prospective validation. With the rapid advancement of CAR-T cell therapies and bispecific antibodies, these approaches are expected to be integrated earlier in the treatment course to improve outcomes in this high-risk population.

### Tandem auto-HSCT and MRD

MRD has been established as a key prognostic indicator in MM, with MRD negativity being strongly associated with prolonged PFS and OS [29]. In a study conducted by Rossi et al., MRD levels were assessed through flow cytometry following the first and second auto-HSCTs. The findings indicated that MRD levels were significantly reduced after the second transplant compared to the first (*p* = 0.005), suggesting that tandem auto-HSCT can further eliminate residual disease and enhance the depth of response [30].

Further evidence from Meera et al. showed that for patients who remained MRD-positive after the first transplant, tandem auto-HSCT significantly reduced MRD levels, with approximately 51% achieving MRD negativity after the second transplant. Notably, the 3-year PFS rate was significantly higher in MRD-negative patients compared to MRD-positive patients (85% vs. 62%, *p* = 0.007), although the difference in OS between the two groups was not statistically significant (97% vs. 91%, *p* = 0.38). Similarly, in patients with HRMM, MRD negativity was associated with improved 3-year PFS (73.7% vs. 49.3%, *p* = 0.007), though OS differences remained statistically insignificant (91.4% vs. 87.6%, *p* > 0.05) [31].

Moreover, a retrospective study indicated that tandem auto-HSCT was associated with a higher MRD negativity rate (81.6%) and an improved 4-year PFS rate of 64.9%, with the greatest benefit observed in patients who achieved CR after the second transplant [32]. Tandem auto-HSCT, when combined with consolidation and maintenance therapy, was associated with improved PFS. Among patients with del(17p), the 5-year OS rate reached 80.2%, compared to 57.1% with single auto-HSCT—a trend consistent with findings from the EMN02/HO95 trial [8].

Notably, in the era of modern quadruplet induction therapy, MRD status remains a critical predictor of treatment response and survival. The GMMG-CONCEPT trial demonstrated that the Isa-KRd regimen (isatuximab combined with carfilzomib, lenalidomide, and dexamethasone) achieved an MRD negativity rate of 67.7% in transplant-eligible high-risk NDMM patients, with over 60% maintaining MRD negativity for at least one year. Median PFS was not reached at a median follow-up of 44 months [33]. Furthermore, the phase III IsKia trial confirmed that Isa-KRd significantly improved MRD negativity rates across all treatment phases. After consolidation, the MRD negativity rate at the 10⁻⁶ threshold was 67% with Isa-KRd, compared to 48% with KRd (*p* < 0.001), with consistent benefit observed in both high-risk and double-hit subgroups [34].

In parallel, the GRIFFIN trial [35] demonstrated that D-RVd combined with single auto-HSCT significantly improved the rate of stringent complete response (sCR) (67% vs. 48%, *p* = 0.0079) and prolonged 4-year PFS (87.2% vs. 70.0%, HR = 0.45, *p* = 0.032). Similarly, the MASTER study [31] demonstrated that patients achieving MRD ≤ 10⁻⁵ after Dara-KRd induction and auto-HSCT could transition directly to a treatment-free observation phase without receiving consolidation or maintenance therapy. Three-year PFS rates were 88% and 79% in the standard- and intermediate-risk groups, respectively, whereas ultra-high-risk patients continued to experience high relapse rates, with a PFS of only 50%, indicating that intensified strategies may still be warranted in HRMM.

MRD negativity serves not only as a marker of deep treatment response but also as a critical tool for prognostic stratification, particularly in high-risk patients. MRD monitoring techniques include next-generation sequencing (NGS), multiparameter flow cytometry (NGF), PET-CT, bone marrow chimerism analysis (e.g., STR typing), and immune repertoire-based PCR assays such as ClonoSEQ [36]. Additionally, the heavy/light chain (HLC) assay is considered a complementary method for MRD evaluation [37].

## Advances in research on tandem auto-HSCT

### Era of conventional chemotherapy

The use of tandem auto-HSCT for treating MM was first documented by Harousseau et al. in 1992 [38]. In 2003, Attal et al. conducted a study showing that tandem auto-HSCT provided significant benefits over single transplantation, particularly in enhancing OS and extending event-free survival (EFS) in patients who did not achieve a very good partial response (VGPR) after the initial transplant [39]. Despite these benefits, the EFS curve showed no enduring plateau, suggesting a continuing risk of relapse following treatment.

Further investigation by Cavo et al. in 2007 revealed that, while OS did not differ significantly between tandem and single auto-HSCT, tandem transplantation led to substantial improvements in CR rate, relapse-free survival (RFS), and EFS. These advantages were particularly evident in patients who did not reach a near-complete response (nCR) following the first transplant [40]. It is important to note that these studies used the VAd regimen (vincristine, doxorubicin, and dexamethasone) for induction therapy. However, since these studies did not stratify patients by cytogenetic risk, survival benefits across different cytogenetic subgroups could not be assessed.

### Era of novel agents

With the introduction of IMiDs and PIs, the preferred induction regimen for MM has shifted to the RVd regimen [41]. In 2013, Cavo et al. conducted a prospective study involving 606 patients with NDMM to assess the efficacy of single auto-HSCT (*n* = 254) versus tandem auto-HSCT (*n* = 352) following bortezomib-based induction therapy. Results demonstrated that PFS was significantly longer in the tandem auto-HSCT group compared to the single transplant group (50 months vs. 38 months, *p* < 0.01), with a higher 5-year OS rate (75% vs. 63%, *p* = 0.02) [42]. These benefits were most evident in patients with high-risk cytogenetic abnormalities who did not achieve CR after bortezomib induction, with the median PFS being 41 months in the tandem transplant group compared to 20 months in the single transplant group (*p* = 0.003). These findings suggest the continued clinical significance of tandem auto-HSCT, even in the context of novel therapies, especially for patients with MM who have suboptimal initial responses.

In 2016, the International Myeloma Working Group (IMWG) recommended sequential tandem auto-HSCT for patients with HRMM following induction therapy with novel agents [43]. The BMT CTN 0702 trial [44, 45] randomly assigned patients, post-induction, into three groups: single auto-HSCT, tandem auto-HSCT, and single auto-HSCT followed by RVd consolidation. After a median follow-up of 38 months, the PFS rates were 53.9%, 58.5%, and 57.8%, respectively, while OS rates were 83.7%, 81.8%, and 85.4%. Although initial results showed no significant differences in PFS or OS between the tandem transplant and single transplant plus RVd consolidation groups, extended follow-up revealed superior outcomes with tandem auto-HSCT for patients with HRMM. Specifically, the 5-year PFS for the tandem auto-HSCT group was 43.7%, compared to 32% for the single transplant group and 37.3% for the RVd consolidation group (*p* = 0.03).

In the EMN02/HO95 phase III randomized trial, the role of tandem auto-HSCT was investigated [8]. After receiving VCd induction therapy (bortezomib, cyclophosphamide, and dexamethasone), patients were randomly assigned to either the VMP regimen (bortezomib, melphalan, and prednisone) or a transplant-based approach, which included both single and tandem auto-HSCT. The trial demonstrated that tandem transplantation provided a superior 5-year PFS rate of 53.5%, compared to 44.9% with single transplantation (HR = 0.74, *p* = 0.036), suggesting improved disease control with tandem transplantation. However, its impact on OS requires cautious interpretation. Notably, among high-risk patients with del(17p), the 5-year OS rate was 80.2% in the tandem transplant group, compared to 57.1% in the single transplant group (*p* = 0.066), suggesting a potential survival benefit for high-risk patients. However, this difference did not reach statistical significance. Moreover, the OS outcomes in this study may have been primarily driven by high-risk patients, as no definitive evidence of OS benefit was observed in standard-risk patients. Therefore, when interpreting the OS results of tandem transplantation, it is essential to consider the differences between risk subgroups to avoid overly broad conclusions for all patient populations.

Moreover, a retrospective study found no significant difference in overall PFS between single and tandem auto-HSCT. However, among patients harboring del(17p), the median PFS in the single transplant group was 40 months, whereas more than half of the patients in the tandem transplant group remained progression-free at 48 months [46]. Similarly, other studies have reported superior PFS outcomes following tandem auto-HSCT in patients with high-risk cytogenetic abnormalities such as t(4;14) and del(17p) [47]. These findings suggest that tandem auto-HSCT confers a significant prognostic advantage in MM patients with adverse cytogenetic features.

### Era of monoclonal antibodies

CD38-targeting monoclonal antibodies (mAbs), such as daratumumab and isatuximab, have become integral components of frontline treatment strategies for MM, significantly enhancing efficacy and playing a crucial role in the management of auto-HSCT.

In transplant-ineligible NDMM patients, the phase III IMROZ trial (NCT03319667) demonstrated that the addition of isatuximab to bortezomib, lenalidomide, and dexamethasone (Isa-VRd) reduced the risk of disease progression or death by 40% (HR = 0.60, 95% CI 0.48–0.75) and significantly improved the MRD negativity rate at a sensitivity threshold of 10^−5^ (50% vs. 28%, *p* < 0.001) [48]. In transplant-eligible patients, the GRIFFIN study (NCT02874742) demonstrated that daratumumab plus VRd (Dara-VRd) achieved a sCR rate of 42.4% at the end of post-transplant consolidation, which further improved to 62.6% after a median follow-up of 49.6 months. Among high-risk subgroups, such as those with gain/amp(1q21), the MRD negativity rate (10^−5^) reached 61.8% [9].

During the maintenance phase following transplantation, the CASSIOPEIA trial indicated that daratumumab maintenance significantly prolonged PFS (not reached vs. 46.7 months in the observation group, HR = 0.53, *p* < 0.0001) and increased the MRD negativity rate among patients achieving at least a complete response (58.6% vs. 47.1%, *p* < 0.0001). However, this benefit was predominantly observed in patients who received VTd induction, whereas no significant difference was noted in those receiving Dara-VTd induction [49].

In addition, the anti-SLAMF7 monoclonal antibody elotuzumab has demonstrated efficacy in RRMM. The ELOQUENT-2 [50] and ELOQUENT-3 [51] trials, both conducted in non-transplant settings, evaluated elotuzumab in combination with lenalidomide (ERd) and pomalidomide (EPd), respectively. Results showed that both combinations significantly prolonged OS compared to their respective control arms (ERd vs. Rd: 48.3 vs. 39.6 months, *p* = 0.0408; EPd vs. Pd: 29.8 vs. 17.4 months, *p* = 0.0217), supporting the clinical value of elotuzumab in combination with IMiDs in transplant-ineligible or relapsed settings.

In NDMM, the clinical efficacy of SLAMF7-targeting monoclonal antibodies remains uncertain. To date, the DSMM-XVII trial [52] is the only trial demonstrating that the addition of elotuzumab to KRd during induction therapy prior to auto-HSCT significantly increased the rate of MRD negativity, suggesting its potential to deepen responses in the frontline setting. However, the GMMG-HD6 (DSMM-HD6) study [53], which evaluated the incorporation of elotuzumab into induction, consolidation, and maintenance therapy with RVd in NDMM patients, showed no significant improvement in PFS or OS compared to standard RVd.

Taken together, while SLAMF7 antibodies such as elotuzumab have established clinical benefit in the relapsed/refractory setting, their role in NDMM remains undefined. Further studies are needed to optimize their timing and integration within contemporary treatment strategies.

Recent studies further suggest that quadruplet induction therapy combined with single auto-HSCT can optimize long-term survival outcomes in HRMM patients. The GMMG-CONCEPT trial evaluated the efficacy of Isa-KRd in transplant-eligible (TE) and transplant-ineligible (TNE) patients with HR NDMM. TE patients underwent six cycles of Isa-KRd induction therapy followed by a single auto-HSCT, achieving An MRD negativity rate of 67.7% after consolidation, which was significantly higher than that in the TNE cohort (54.2%). At a median follow-up of 54 months, the four-year PFS rate for the TE group reached 59.4% [33], markedly superior to historical data in HRMM patients, where three-year PFS typically falls below 50% [54].However, for ultra-high-risk patients, such as those with del(17p) or amp(1q21), while single auto-HSCT combined with monoclonal antibodies can extend PFS, it remains insufficient to fully overcome their adverse prognosis.

Against this backdrop, the strategy of incorporating tandem auto-HSCT with monoclonal Antibodies has emerged as An area of significant interest in HRMM. The IFM 2018−04 phase II trial evaluated the feasibility of Dara-KRd induction/consolidation with tandem auto-HSCT in HR TE-NDMM patients. Among 50 enrolled patients, 72% successfully completed tandem auto-HSCT, and the pre-maintenance MRD negativity rate (NGS, 10^−6^) reached 94%. After a median follow-up of 32 months, the 24-month PFS was 87%, and the OS was 94%, suggesting substantial survival benefits with this intensified approach [55]. However, despite these promising outcomes, some ultra-high-risk patients (e.g., del(17p)) still experienced disease progression, indicating that monoclonal antibodies combined with tandem auto-HSCT, while effective, may not be sufficient to completely mitigate the adverse impact of ultra-high-risk cytogenetics. Therefore, further investigation is warranted to explore more innovative treatment strategies, such as CAR-T therapy, bispecific antibodies (BsAbs), or novel targeted agents, to further enhance long-term survival outcomes in UHRMM patients.

### A new era in immunotherapy: CAR-T cell therapy and bispecific antibodies

Chimeric antigen receptor T-cell (CAR-T) therapy utilizes gene engineering techniques to introduce genes encoding CARs into T cells, enabling them to precisely recognize tumor-associated antigens and enhance cytotoxic activity against malignant cells [56]. In 2016, Ali et al. first reported that BCMA-targeted CAR-T therapy demonstrated promising antitumor activity and manageable safety in patients with RRMM [57]. Since then, CAR-T therapy has rapidly advanced, with Abecma (idecabtagene vicleucel) becoming the first FDA-approved BCMA-targeted CAR-T product for RRMM in March 2021 [58]. Beyond BCMA, novel targets such as CS1, GPRC5D, CD38, and CD138 are under investigation in clinical trials, expanding the therapeutic landscape of CAR-T in MM.

Currently, clinical data on the combination of CAR-T therapy with tandem auto-HSCT in MM remain limited. However, CAR-T therapies have shown substantial survival benefits across multiple trials [59, 60]. Garfall et al. [61] evaluated the use of CD19-targeted CAR-T cells (CTL019) following high-dose melphalan and auto-HSCT in 10 patients with R/R MM. A total of 50 × 10^6^ CTL019 cells were infused post-auto-HSCT, with a median PFS of 185 days (range, 42–479), and two patients achieved longer PFS compared to their prior single auto-HSCT, providing early clinical support for this combinatorial strategy.

Among all CAR-T therapies, BCMA-targeted products remain the most extensively studied and widely applied. Long-term follow-up from the CARTITUDE-2 trial [62] demonstrated the robust efficacy of ciltacabtagene autoleucel (cilta-cel) in earlier lines of therapy. In cohort A (1–3 prior lines, lenalidomide-refractory), with a median follow-up of 29.9 months, the MRD negativity rate at 10⁻⁵ was 100%, the ORR was 95%, and 90% achieved CR or better. Two-year PFS and OS rates were 75%. In cohort B (early relapse), ORR reached 100%, CR rate was 90%, and 2-year PFS and OS were 73% and 84%, respectively. CAR-T–related toxicities were manageable, with no new safety signals observed. These results support the potential of cilta-cel to deliver deep and durable responses in both early refractory and relapsed settings, paving the way for its use in frontline therapy.

In parallel, BsAbs have emerged as a major breakthrough in MM immunotherapy. BsAbs simultaneously bind a tumor antigen—such as BCMA—and CD3 on T cells, promoting direct T-cell–mediated cytotoxicity [63]. Teclistamab became the first FDA-approved BsAb for RRMM in October 2022, for patients who had received ≥ 4 prior lines of therapy [64]. In the MajesTEC-1 study [64], teclistamab achieved an ORR of 63%, a CR rate of 39%, with median PFS and OS of 11.3 and 18.3 months, respectively. Similarly, the MagnetisMM-3 study [65] reported an ORR of 61.0% and CR rate of 35% with elranatamab, with 15-month PFS and OS rates of 50.9% and 56.7%, respectively.

CAR-T cell therapy and BsAbs each have distinct features in MM management. CAR-T offers the potential for deep remission and prolonged treatment-free intervals with a one-time infusion [56]. However, its manufacturing process takes 4–6 weeks, requires specialized transplant centers, and may not be feasible for patients with rapidly progressing disease or production failure. In contrast, BsAbs are off-the-shelf agents that allow prompt treatment initiation and are more suitable for patients who are ineligible for or unable to wait for CAR-T. Nonetheless, BsAbs require continuous administration, potentially increasing long-term toxicity burden. Fixed-duration treatment strategies (e.g., under investigation in follow-up analyses of MajesTEC-1) are being actively explored.

As studies like KarMMa-3 [66] and CARTITUDE-4 [67] continue to advance CAR-T therapy into earlier treatment settings, BsAbs are also being increasingly incorporated into combination regimens. Future treatment strategies should be tailored to individual patient profiles, taking into account disease characteristics, prior therapies, treatment accessibility, and patient preferences, in order to maximize clinical benefit.

## Controversies surrounding tandem auto-HSCT

While some studies have posited that patients with HRMM or those achieving a VGPR after the first auto-HSCT might benefit from tandem auto-HSCT, its clinical efficacy remains a subject of ongoing controversy [68]. This controversy largely stems from heterogeneity in trial designs, variations in induction regimen intensity, maintenance strategies, and risk stratification approaches. Therefore, a systematic evaluation of existing evidence is warranted to clarify the true role of tandem auto-HSCT within the context of modern therapeutic paradigms and to provide a more precise basis for clinical decision-making.

In July 2024, the FDA approved Dara-VRd as an induction and consolidation regimen for patients with NDMM, marking the formal establishment of CD38 monoclonal antibodies as a cornerstone of frontline therapy in transplant-eligible patients. As quadruplet regimens increasingly become the standard of care for induction, deep responses can often be achieved with a single auto-HSCT, thereby challenging the clinical value of the traditional tandem auto-HSCT approach.

Early randomized controlled trials, such as GMMG-HD2 [69], and related meta-analyses predominantly [70] employed conventional triplet induction regimens such as VCD (bortezomib, cyclophosphamide, dexamethasone) or TD (thalidomide, dexamethasone), which do not capture the therapeutic efficacy of modern combinations incorporating CD38 monoclonal antibodies with PIs and IMiDs. In the GMMG-HD2 trial, although the tandem auto-HSCT group achieved a higher CR rate, there were no significant differences between the two arms in OS or EFS, suggesting that tandem auto-HSCT did not confer a clear long-term survival benefit under contemporary treatment conditions [69]. Similarly, meta-analyses have shown that while tandem transplantation may improve response rates, it does not significantly enhance EFS or OS and is associated with a higher treatment-related mortality, potentially offsetting the benefits of deeper remission [70]. It is important to note that the studies included in this meta-analysis were published before 2008, preceding the widespread use of novel agents, and lacked stratification by high-risk cytogenetic features such as del(17p), thereby limiting the relevance of the findings to contemporary practice and to high-risk subgroups like HRMM.

A retrospective study by Venner et al. in Canada included 381 patients with HRMM, all of whom received VCD induction therapy followed by auto-HSCT and maintenance with IMiDs and/or PIs. The results showed no significant differences between the single and tandem auto-HSCT groups in terms of ORR (98.3% vs. 98.6%, *p* = 0.19), ≥VGPR rate (90.5% vs. 89.9%, *p* = 0.86), PFS(35.2 vs. 35.3 months, *p* = 0.88), or OS(92.6 vs. 88.9 months, *p* = 0.72) [71]. However, the retrospective nature of the study introduces the possibility of selection bias. The single transplant group may have included patients with more favorable baseline prognostic features, while the tandem transplant group might have been enriched for individuals with higher disease burden. Therefore, caution is warranted when interpreting these findings.

Multiple contemporary studies have demonstrated that intensified induction therapy alone can achieve deep responses, thereby diminishing the reliance on tandem auto-HSCT. The GMMG-CONCEPT study demonstrated that in patients with HRMM, durable disease control could be achieved with a single auto-HSCT following induction with the quadruplet regimen Isa-KRd. The efficacy observed was comparable to that historically seen with tandem transplantation, highlighting the potential of intensified induction to reduce the need for sequential transplants [33]. The CASSIOPEIA [72]and PERSEUS [73] studies have respectively confirmed that, in the setting of Dara-VTd or Dara-VRd used for induction, consolidation, and CD38 monoclonal antibody–based maintenance, a single auto-HSCT is sufficient to achieve superior long-term PFS and high rates of MRD negativity. These findings further diminish the marginal benefit of tandem transplantation in the context of modern, intensified therapeutic strategies.

Maintenance therapy strategies represent another key factor influencing the value of tandem auto-HSCT. Early studies often did not incorporate long-term maintenance or relied solely on lenalidomide monotherapy. In contrast, contemporary treatment approaches increasingly utilize dual-agent maintenance combining CD38 monoclonal antibodies with IMiDs, which has been shown to further deepen responses following a single auto-HSCT and reduce the reliance on tandem transplantation.

Although most studies have failed to demonstrate a clear long-term survival benefit of tandem auto-HSCT in patients with HRMM, recent retrospective data suggest that, in the context of triplet or quadruplet induction and modern maintenance strategies, a subset of HRMM patients may still achieve deeper responses with tandem auto-HSCT—reflected by a higher rate of deep remission (81.8% vs. 63.2%) and an approximate 43.7% reduction in the 3-year risk of disease progression [32]. However, for patients with uHRMM characterized by multiple high-risk cytogenetic abnormalities—such as 1q21 gain/amplification, del(17p), t(4;14), or t(14;16)—PFS and OS remain significantly inferior even with tandem transplantation (HR = 2.81 compared to general HRMM) [74]. These findings suggest that conventional intensification strategies are insufficient to overcome the aggressive biology of uHRMM, underscoring the need to prioritize the integration of novel immunotherapies such as CAR-T cells or BsAbs.

In summary, the applicability of tandem auto-HSCT should be guided by individualized decision-making based on patient risk profile, treatment response, and available therapeutic options. Among patients with HRMM, tandem auto-HSCT may be considered when CR or MRD negativity is not achieved after a single auto-HSCT and when CD38 monoclonal antibody–based maintenance is not feasible. For patients with uHRMM, who often exhibit limited responsiveness to conventional intensification strategies, priority should be given to integrating novel immunotherapies such as CAR-T cells or BsAbs. In patients with poor performance status or intolerance to high-dose chemotherapy, optimizing induction and maintenance regimens should take precedence over pursuing tandem auto-HSCT.

**Fig. 2:**
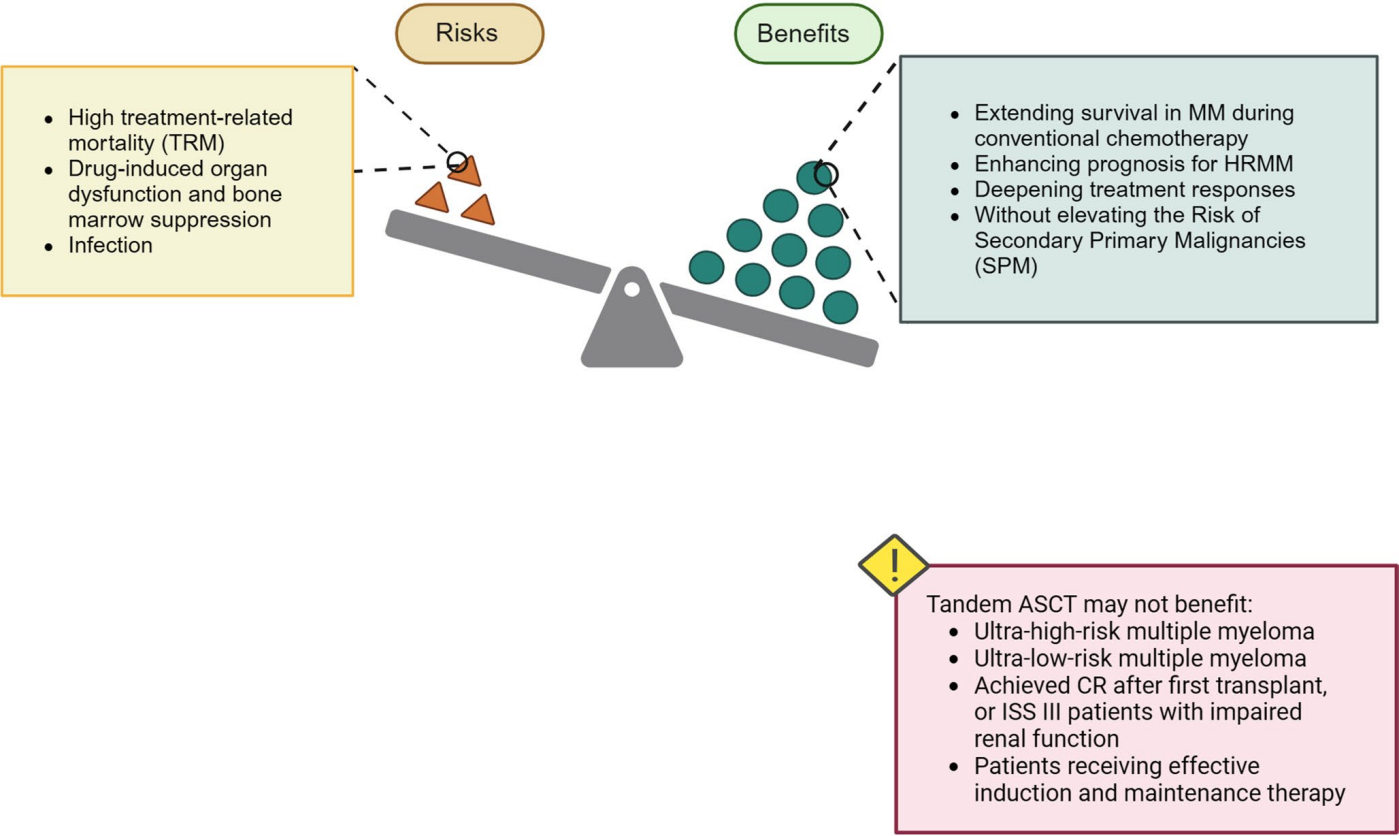
Benefits and risks of tandem auto-HSCT. While tandem auto-HSCT can deepen responses and prolong survival, especially in high-risk patients, its benefit is not consistent across all risk groups. Notably, ultra–low-risk patients and those achieving deep remission after the first transplant may not derive additional benefit from tandem auto-HSCT. Therefore, individualized transplant strategies based on risk stratification and treatment response are warranted. Abbreviations: TRM: Treatment-related mortality; MM: Multiple myeloma; HRMM: High-risk Multiple myeloma; SPM: Secondary Primary Malignancy. Created with BioRender.com

In conclusion, although tandem transplantation may offer remission benefits in specific cases, its broad applicability requires careful evaluation of the balance between its potential efficacy and the associated risks. This calls for personalized approaches based on patient-specific risk profiles, disease characteristics, and response to initial therapies (Fig. 2; Table 1).

**Table 1:** Comparative analysis of single and tandem auto-HSCT studies

Source	Induction therapy	Definition of High-Risk MM	High-Risk MM (No. and %) (sin vs. tan)	No. of patients (sin vs. tan)	CR plus VGPR (%) (sin vs. tan)	PFS, Median (sin vs. tan)	*p* value for PFS	OS, Median (sin vs. tan)	*p* value for OS
Attal 2003 [39]	VAd	NR	NR	199 vs. 200	42 vs. 50	25 vs. 30mo	0.03	48 vs. 58mo	0.01
Cavo 2007 [40]	VAd	NR	NR	163 vs. 158	33 vs. 47	23 vs. 35mo	0.001	46 vs. 43mo	0.9
Fermand 2009 [70]	VAd + MLP	NR	NR	112 vs. 113	35 vs. 37	NR	0.61	NR	0.6
Cavo 2013 [42]	Bor-based	NR	NR	254 vs. 352	NR	38 vs. 50mo	< 0.001	5y: 67% vs. 75%	0.002
Mai 2016 [69]	VAD/VID/VCAP	NR	NR	188 vs.197	16%(CR) vs. 19.4%(CR)	25 vs. 28.7mo (EFS)	0.53	73 vs.75.3 mo	0.33
Stadtmauer 2019 [44]	Len-based/dex-based	β2-microglobulin > 5.5 mg/L or presence ≥ 1 of the HRCAs: t(4;14), t(14;16), t(14;20), del(17p), del(13) by conventional cytogenetics	75 (29%) vs. 72 (29%)	257 vs. 247	44 vs. 50	38mo: 53.9% vs. 58.5%	> 0.05	38mo: 83.7% vs. 81.8%	> 0.05
Cavo 2020 [8]	VCd	Presence ≥ 1 of the HRCAs: del(17p), t(4;14), or t(14;16)	42 (25.1%) vs. 39 (21.8%)	209 vs. 210	NR	5y: 44.9% vs. 53.5%	0.036	5y: 72.6% vs. 80.3%	0.022
Villalba 2022 [46]	VTd/VRd	Presence ≥ 1 of the HRCAs: del17p, t(4;14), t(14;16) or 1q21+	32 (22.6%) vs. 32 (45.1%)	142 vs. 71	29.6 vs. 36.6	41 vs. 48mo	0.33	58% vs. 78%	0.18
Venner 2024 [71]	Bor-based	Presence ≥ 1 of the HRCAs: del(17p), t(4;14), or t(14;16)	High	242 vs. 139	90.5 vs. 89.9	35.2 vs. 35.3mo	0.88	92.6 vs. 88.9mo	0.72
Xuelin Dou 2025 [74]	RVD/VCD/PDD/VTD/Daratumumab-based quadruplet	Presence ≥ 2 of the following—ISS Ⅲ, LDH↑, 1q21+, del(17p), t(4;14), t(14;16), or hypodiploidy; cPCs ≤ 4%; EME; Suboptimal early response	High	57 vs. 55	91.3 vs.94.5	58.1% vs. 64.7%	0.064	3y:79.1% vs. 79.5%	0.432

*Bor* Bortezomib; *CR* Complete remission; *cPCs* circulating Plasma Cells; *dex* Dexamethasone; *EME* Extramedullary extraosseous disease; *HRCAs* High-Risk Cytogenetic Abnormalities; *Len* Lenalidomide; *LDH* Lactate Dehydrogenase; *MLP* Melphalan; *MM* Multiple myeloma; *mo*: Month; *NR* Not reported; *OS* Overall Survival; *PFS* Progression-Free Survival; *PDD* bortezomib-liposomal doxorubicin-dexamethasone; *sin* Single autologous stem cell transplantation; *tan* Tandem autologous stem cell transplantation; *VAd* Vincristine-Adriamycin-dexamethasone; *VCd* Bortezomib-cyclophosphamide-dexamethasone; *VID* vincristine, idarubicin, dexamethasone; *VCAP* vincristine, cyclophosphamide, doxorubicin, prednisone; *VGPR* Very good partial response; *VRd* Bortezomib-lenalidomide-dexamethasone; *VTd* Bortezomib-thalidomide-dexamethasone; *vs*. Versus; *y* Year. 1q21+: 1q21 gain/amplification

## Sequential auto-HSCT followed by Allo-HSCT

Unlike auto-HSCT, allo-HSCT involves the transfer of hematopoietic stem cells from a healthy donor to the recipient, aiming to reconstitute the patient’s hematopoietic and immune systems. Allo-HSCT is considered the only potentially curative treatment option for MM, primarily due to its graft-versus-myeloma (GVM) effect. However, compared to other hematologic malignancies, the GVM effect in MM is relatively modest, limiting its clinical efficacy in fully eradicating residual myeloma cells [75, 76]. Moreover, its clinical application is hampered by significant challenges, including treatment-related mortality and graft-versus-host disease (GVHD).

Studies investigating the efficacy of sequential auto-allo therapy have yielded inconsistent results. Data from the IFM trial indicated no significant differences in PFS or OS between tandem auto-HSCT and auto-allo HSCT for patients with HRMM [77]. Conversely, research by Kröger et al. demonstrated a significantly lower risk of relapse or disease progression in the auto-allo group compared to the tandem auto-HSCT group (44% vs. 77%, *p* = 0.002). However, these differences did not translate into statistically significant improvements in either PFS or OS [78].

The BMT CTN 0102 trial evaluated outcomes in standard-risk and HRMM patients undergoing either tandem autologous hematopoietic stem cell transplantation (auto-auto) or autologous followed by reduced-intensity allogeneic transplantation (auto-allo). Among standard-risk patients, no significant differences were observed between the two arms in 6-year PFS (25% vs. 22%, *p* = 0.32) or OS (60% vs. 59%, *p* = 0.85). In the high-risk group, although the auto-allo arm was associated with a higher non-relapse mortality (NRM; 43%), it demonstrated a significantly lower 6-year relapse rate (31% vs. 77%, *p* = 0.005) and improved PFS (31% vs. 13%, *p* = 0.05), while OS remained comparable between groups (47% vs. 51%, *p* = 0.69) [79] These results suggest that auto-allo hematopoietic cell transplantation (HCT) may provide superior disease control in high-risk patients, though its clinical application warrants further investigation.

In patients with MM and specific high-risk cytogenetic markers, such as del(17p) and/or t(4;14), auto-allo HSCT may offer superior long-term outcomes. Gagelmann et al. found that in newly diagnosed patients with MM and these abnormalities, both tandem auto-HSCT and auto-allo approaches provided greater 5-year PFS compared to single auto-HSCT. Auto-allo was particularly effective in reducing relapse rates and improving survival [80]. Similarly, Björkstrand et al. reported a significantly higher 60-month PFS in the auto-allo group compared to the tandem auto-HSCT group (39% vs. 19%, *p* = 0.004), along with a lower risk of relapse or progression (43% vs. 78%, *p* = 0.001). Regarding OS however, no significant difference was observed between the groups (63% vs. 60%, *p* = 0.753). Among patients with del(13) abnormalities, auto-allo HSCT demonstrated notable advantages in both PFS and OS [81]. The DSMM V trial specifically compared outcomes in patients having MM with del(13q) abnormalities treated with auto-allo versus tandem auto-HSCT. The results demonstrated that the median PFS was significantly longer in the auto-allo group compared to the tandem auto-HSCT group (34.5 months vs. 21.8 months, *p* = 0.003), although the 2-year NRM was also notably higher [82]. With recent advancements in genetic understanding and updated definitions of HRMM, the prognostic value of del(13)/del(13q) has been reassessed and is no longer classified as a definitive high-risk marker.

Although auto-allo transplantation has advantages in reducing relapse rates, its broader clinical use is constrained by high transplant-related mortality, GVHD, and significant treatment costs. Although auto-allo HSCT may provide improved long-term survival for select patients with HRMM, these limitations restrict its widespread use in clinical settings. Consequently, auto-allo transplantation is primarily confined to clinical trials focusing on patients with HRMM (Table 2) [84, 85].

**Table 2:** Comparative analysis of Auto-auto and Auto-allo studies

Source	Induction therapy	Definition of High-Risk MM	High-Risk MM (No. and %) (tan vs. auto-allo)	No. of patients (tan vs. auto-allo)	CR plus VGPR (%) (tan vs. auto-allo)	PFS, Median (tan vs. auto-allo)	*p* value for PFS	OS, Median (tan vs. auto-allo)	*p* value for OS	NRM (%) (tan vs. auto-allo)	*p* value for NRM
Garban 2006 [77]	VAd	β2-microglobulin > 3 mg/L and del(13q) at diagnosis by FISH	54 (83%) vs. 193 (88%)	219 vs. 65	51 vs.62.2	30 vs. 25mo	0.56	41 vs. 35mo	0.27	5 vs. 10.87	NR
Björkstrand 2011 [81]	VAd	del (13q) bu FISH	del (13): 29 (46%) vs. 63 (42%)	104 vs. 91	44 vs. 56	60 mo: 39% vs. 19%	0.004	63% vs. 60%	0.753	60mo: 4 vs. 16	< 0.001
Krishnan 2011 [83]	Thalidomide and dex	NR	48 vs. 37	436 vs. 189	71% vs. 68%	3y: 46% vs. 43%	0.671	3y:80% vs. 77%	0.191	NR	NR
Knop 2019 [82]	VAd-based	del(13q) by FISH	High	73 vs. 126	30.9 vs. 58.6	21.8 vs. 34.5mo	0.003	71.8 vs. 70.2 mo	0.856	2y: 4.1 vs. 14.3	0.008
Giralt 2020 [79]	Bor-based/Thal-based	-	Standard	436 vs. 189	42 vs. 42	6y: 25% vs. 22%	0.32	60% vs. 59%	0.85	11 vs. 20	< 0.001
		β2-microglobulin ≥ 4 mg/dl or del(13) by conventional cytogenetics	high	48 vs. 37	6 vs. 32	6y: 13% vs. 31%	0.07	47% vs. 51%	0.69	11 vs. 22	0.17
Kröger 2023 [78]	Bor-based/dex-based	t(4;14) or del(17p) by FISH	8 (17.39%) vs. 24 (18.18%)	46 vs. 132	20 vs. 36	124mo: 21% vs. 43%	0.10	50% vs. 52%	0.86	2 vs. 13	0.04

*auto* Autologous stem cell transplantation; *allo* Allogeneic hematopoietic stem cell transplantation; *Bor* Bortezomib; *CR* Complete remission; *dex* Dexamethasone; *mo* Month; *MM* Multiple myeloma; *NR* Not reported; *NRM* Non-Relapse Mortality; *OS* Overall Survival; *PFS* Progression-Free Survival; *Thal* Thalidomide; *tan* Tandem autologous hematopoietic stem cell transplantation; *VGPR* Very good partial response; *VAd* Vincristine-adriamycin-dexamethasone; *vs.* Versus; *y* Year

With the rapid development of novel therapies, HRMM patients who fail to achieve MRD negativity after the first transplant face a critical treatment decision—whether to undergo a second transplant or opt for innovative consolidation strategies such as CAR-T therapy or bispecific antibodies. This choice requires a comprehensive evaluation of efficacy, safety, and accessibility.

## Tandem auto-HSCT and novel consolidation therapies

In the context of quadruplet therapy (such as the D-RVd regimen) combined with auto-HSCT becoming the standard treatment for NDMM, high-risk patients who fail to achieve MRD negativity after the first transplant require a more refined selection of consolidation strategies. The CASSIOPEIA and PERSEUS trials showed that D-RVd induction followed by transplantation resulted in a four-year PFS of 87.2% in standard-risk patients [72, 73], while high-risk patients achieved only 64.8%, indicating the need for more intensive consolidation.

Traditionally, tandem transplantation enhances MRD negativity rates by increasing melphalan dosing. The six-year follow-up of the STAMINA trial showed that in high-risk patients who had not received quadruplet induction, the six-year PFS was 43.6% (vs. 26% for single transplantation, *p* = 0.03), but OS showed no significant improvement. Additionally, rates of grade ≥ 3 infections, mucositis, and treatment-related mortality were 38%, 28%, and 3.8%, respectively, indicating significant toxicity. ITT analysis showed no OS difference, suggesting that the benefits of tandem transplantation are mainly limited to specific high-risk subgroups [44]. Therefore, in the era of modern quadruplet induction, patient selection for tandem transplantation should be precisely stratified, balancing toxicity risks and survival benefits.

In comparison, cilta-cel and bispecific antibody (teclistamab) demonstrate a superior risk-benefit profile. The CARTITUDE-1 trial showed that in RRMM patients who failed quadruplet therapy and received cilta-cel, the median PFS was not reached, with a 27-month PFS rate of 54.9%, including 64.2% in sCR patients and 73.0% and 78.8% in those with MRD negativity for ≥ 6 months and ≥ 12 months, respectively, with an MRD negativity rate (10^−5^) of 92% [86]. For patients with persistent clonal residual disease after the first transplant, CAR-T therapy effectively eliminates malignant plasma cells, potentially preventing disease progression more efficiently. The MajesTEC-1 study reported that teclistamab treatment for RRMM achieved An ORR of 76.9%, ≥CR rate of 57.7%, 12-month PFS rate of 68%, and median PFS of 11.3 months, with 93.3% of ≥ CR patients achieving MRD negativity. The ≥ 3 grade infection rate was 73.1%, and CRS incidence was 96.2% (none ≥ 3 grade) [87]. Its subcutaneous administration allows for convenient outpatient management, making it particularly suitable for patients with non-clonal residual disease or those intolerant to the toxicity of tandem transplantation.

The adoption of novel therapies is limited by high costs and resource constraints, with CAR-T therapy being expensive and requiring a long manufacturing process, while bispecific antibodies, though slightly more affordable, remain inaccessible in some regions. Therefore, optimizing tandem transplantation remains a viable option. Future research should explore improved sequential strategies, such as post-transplant bridging with CAR-T or quadruplet induction followed by bispecific antibody maintenance, to enhance efficacy while reducing toxicity and refining the treatment landscape for HRMM.

## Conclusion

In recent years, the treatment landscape of MM has undergone a profound transformation. With continuous advancements in immunotherapy, the long-standing central role of auto-HSCT is facing unprecedented challenges. The growing adoption of quadruplet induction regimens, combination maintenance strategies, and MRD-guided treatment approaches has significantly deepened responses following a single auto-HSCT, leading to a gradual decline in the marginal benefit of tandem auto-HSCT [12].

The EMN02/HOVON95 study [88] demonstrated that the response to intensive treatment strategies varies significantly across molecular and clinical risk strata in MM. High-risk patients, particularly those with adverse cytogenetics, derived substantial benefit from high-dose melphalan followed by tandem auto-HSCT. In contrast, ultra–low-risk patients showed no additional survival benefit from auto-HSCT compared to standard therapy, supporting a risk-adapted approach.

Furthermore, a large-scale retrospective analysis from the German DSMM registry indicated that tandem auto-HSCT significantly improved overall survival in patients who failed to achieve complete remission after induction or after the first auto-HSCT [89]. However, for those who had already achieved deep remission (CR or MRD-negative status) following the first transplant, or who had ISS stage III disease with renal impairment, a single transplant appeared to be sufficient and less toxic. These findings collectively highlight that the benefit of tandem auto-HSCT is not universal, and its utility should be guided by refined risk stratification, early treatment response, and functional status. In the era of potent induction regimens and maintenance therapies, personalized transplant strategies are increasingly important to optimize long-term outcomes while minimizing unnecessary toxicity (Fig.2).

The emergence of BsAbs has also challenged the traditional role of auto-HSCT. Results from trials such as ASH GMMG HD10/DSMM XX [90] and EMN30 [91] have shown that BsAbs can achieve 100% MRD negativity post-ASCT, significantly reducing the necessity for a second transplant. Furthermore, the PERSEUS trial demonstrated that the combination of Dara-VRd with a single auto-HSCT can deliver PFS exceeding 10 years, reinforcing the therapeutic value of single auto-HSCT [73] and raising questions about the continued need for tandem auto-HSCT in the modern treatment era.

With the advent of emerging therapies, allo-HSCT has gradually receded from mainstream clinical practice due to its associated toxicity and procedural complexity, now largely confined to select clinical trials. These novel immunotherapies not only enhance efficacy but also offer improved safety profiles, positioning themselves as strong alternatives to auto-HSCT.

Looking ahead, the ability to accurately identify patient subgroups suited for different therapeutic strategies and to optimize the integration of auto-HSCT with novel immunotherapies will become a central focus of research. Advances in dynamic MRD monitoring provide critical support for assessing treatment response and guiding retreatment decisions, paving the way for a shift from fixed treatment algorithms to response-adapted, individualized, risk-based approaches.

Preliminary studies have shown that combining auto-HSCT with CAR-T cell therapy in patients with high tumor burden or extramedullary disease can significantly enhance depth of response and prolong PFS [73], with favorable safety and feasibility, offering a practical foundation for integrated treatment strategies.

In the future, identifying optimal candidates for various treatment modalities and refining the sequencing and combination of auto-HSCT with immune-based therapies will be key to maximizing clinical benefit. For patients with high-risk or ultra-high-risk disease, a multi-phase therapeutic approach—targeting tumor debulking, hematopoietic reconstitution, and microenvironmental control—through the combined use of tandem auto-HSCT and immunotherapy holds promise for achieving deeper disease control and sustained survival outcomes.

## Data Availability

No datasets were generated or analysed during the current study.
